# Anterior Cruciate Ligament Return to Play: “A Framework for Decision Making”

**DOI:** 10.3390/jcm14072146

**Published:** 2025-03-21

**Authors:** Roberto Ricupito, Alberto Grassi, Firas Mourad, Luigi Di Filippo, Massimiliano Gobbo, Filippo Maselli

**Affiliations:** 1 Restart Physiotherapy, 00162 Rome, Italy; 2 Education and Research Department, Isokinetic Medical Group, FIFA Medical Centre of Excellence, 40132 Bologna, Italy; alberto.grassi@ior.it; 3 2nd Clinica Ortopedica e Traumatologica, Istituto Ortopedico Rizzoli IRCCS, 40136 Bologna, Italy; 4 Department of Health, LUNEX University of Applied Sciences, 4671 Differdange, Luxembourg; firas.mourad@me.com; 5 Luxembourg Health & Sport Sciences Research Institute A.s.b.l., 4671 Differdange, Luxembourg; 6 FisioAnalysis Mædica, 15121 Alessandria, Italy; fisioanalysis@gmail.com; 7 Department of Human Neurosciences, Sapienza University of Rome, 00185 Rome, Italy; masellifilippo76@gmail.com; 8 Department of Clinical and Experimental Sciences, Neuroscience Unit, University of Brescia, 25123 Brescia, Italy; massimiliano.gobbo@unibs.it; 9 Sovrintendenza Sanitaria Regionale Puglia INAIL, 70126 Bari, Italy

## 1. Introduction

Anterior cruciate ligament (ACL) injury is common in athletic individuals and often leads to physical impairments, a low rate of return to performance, reinjuries, and sometimes reductions in career length [1,2,3].

In soccer, the average return to play (RTP) rate ranges between 72% and 90%, while the return-to-performance rate varies between 53% and 80% across different studies. The mean post-injury career length typically ranges from 3 to 8 years; however, it is significantly shorter than that of a control group of healthy athletes, with an average reduction of 1 to 2 years. Additionally, a decline in performance has been observed across various sports populations following injury [3,4,5,6,7,8,9]. Surgical reconstruction of the anterior cruciate ligament (ACLR) is an increasingly common treatment for ACL injuries, with the incidence in the United States rising from 40.9 per 10,000 patients in 2004 to 47.8 per 10,000 in 2009 [10].

The objective of surgery and rehabilitation is to return to the field as soon as possible while minimizing the risk of reinjuries, which range from 5 to 40% between ipsilateral and contralateral rupture, with the greatest portion of reinjuries occurring during the first 2 years post-ACLR [6,11,12,13,14]. The risk factors associated with reinjury vary from articular geometry, surgery errors, young age, familiarity, sex (conflicting evidence), functional factors, and a return to pivoting [12,15,16,17,18,19].

The most frequent cause of reinjuries is a second traumatic event, followed by technical errors, biological failure, and a combination of factors.

**Figure 1:**
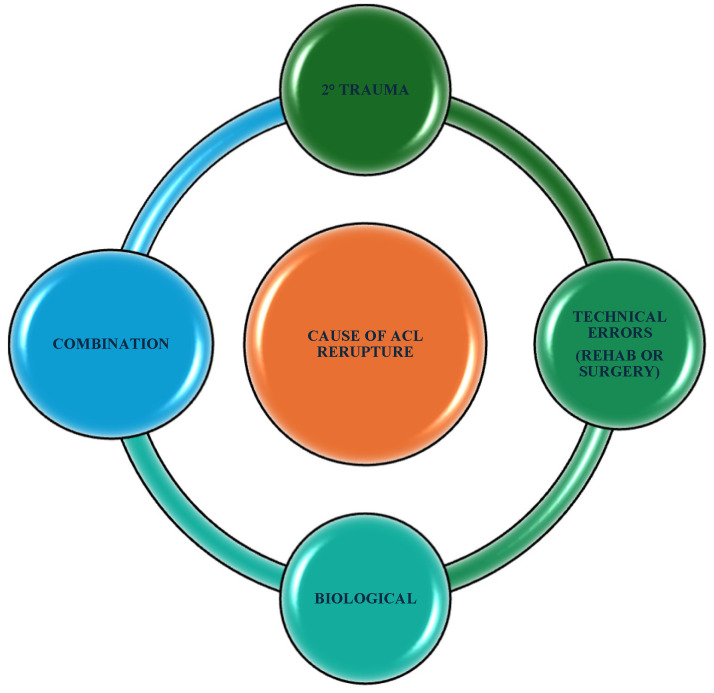
Principal factors correlated with ACL revision.

The first refers to a new trauma (contact or non-contact injuries); the second to tunnel malposition, error in graft placement or selection, or non-treated meniscal or other ligament injury; the third to host rejection or infection; and the fourth to a combination of all these factors [20] (Figure 1).

This literature review indicates that the average RTP time after anterior ACLR is approximately 8.7 months, aligning with recent findings from Hong et al., who reported an RTP range of 6.1 to 11.1 months for soccer players [21]. However, while 8.7 months represents a significant absence from soccer matches, it is shorter than the recovery times observed in other sports and among non-professional athletes [22]. For instance, NBA players have an average RTP of 9.8 months, while NFL athletes take around 12.6 months. Among recreational athletes, the recovery period can extend up to 33.7 months [23,24]. Additionally, studies show that amateur and recreational soccer players experience longer RTP times [21]. One factor that negatively affects RTP is the need for revision ACL surgery. Furthermore, the scientific literature provides strong evidence that a fear of reinjury and knee-related issues are among the most common reasons why athletes do not return to their sport [21,22,23,24].

As previously stated, despite the high pre/postoperative expectations of patients, not everyone returns to their sport at their preinjury level [25].

RTP following ACLR is influenced by a combination of physical, psychological, and rehabilitation-related factors. Younger athletes and those with higher subjective IKDC scores demonstrate a greater likelihood of RTP [14]. Additionally, high compliance with structured postoperative rehabilitation, including jumping and agility exercises, is associated with a higher RTP rate, highlighting the critical role of rehabilitation in optimizing recovery [26]. Consequently, specialists and physical therapists should emphasize comprehensive rehabilitation protocols focusing on neuromuscular strength and function to facilitate a successful RTP.

From a predictive standpoint, three key factors consistently correlate with RTP: the athlete’s goal of returning to their preinjury sport levels, psychological readiness, and ACL injury on the non-dominant leg [27].

In terms of surgical outcomes, 70 to 90% of athletes (non-professional and level 1) successfully returned to at least one game post-ACLR, with an average RTP time of 10.4 months [5,14]. However, the presence of concomitant medial collateral ligament injuries was identified as a significant negative predictor for RTP, while meniscal injuries were associated with reduced RTP rates and career longevity, although without statistical significance [5]. Additionally, a substantial proportion of ACL injuries occurred early in the season, underscoring the need for enhanced preventive strategies to mitigate injury risk [5,14,27].

One of the key aspects in the RTP pathway is the role of clinical and functional tests, used across different studies, to determinate if an athlete is ready to return to performance or if there is only a minor risk of sustaining a 2° injury. The RTP test likely helps us understand this readiness better from a “functional” perspective, but it has a low capacity for identifying individuals at risk [28].

Return to play following ACLR is a multifaceted process that extends beyond merely passing functional tests. Evidence suggests that RTP test performance is not significantly associated with a reduced risk of reinjury, calling into question the predictive validity of these assessments [29]. Studies indicate that passing the RTP criteria does not substantially decrease the reinjury risk, as reinjury rates remain similar between those who pass and those who fail these tests. However, passing these tests is associated with a higher RTP rate [25,30].

Another critical issue is the low pass rate of the RTP criteria. At six months post-ACLR, only 3% of patients successfully meet all criteria, and this number increases only modestly to 11% at nine months [31]. Quadricep strength deficits persist as a major barrier, while self-reported knee function also shows insufficient improvement over time [32]. Even after extended rehabilitation, a substantial proportion of athletes fail to meet the RTP benchmarks, highlighting concerns about the applicability of current testing protocols. Compounding this issue, many healthy individuals fail RTP tests, with 5.3% to 42.2% failing hop tests and up to 50% failing strength assessments. Additionally, an asymmetry index ≥10% was found in several strength tests, suggesting that this cutoff may not be entirely realistic [33]. These findings highlight the need for more practical and evidence-based RTP criteria to avoid unnecessary restrictions while ensuring safe RTP [33]. Many healthy individuals without a history of ACL injury also fail RTP tests, with failure rates ranging from 5% to 42% across different functional assessments [26]. These findings suggest that the existing RTP thresholds may be either too restrictive or insufficiently representative of a safe RTP [33].

The criticism of the current RTP tests extends beyond their predictive capacity to their clinical practicality and validity [25,32,33]. Many tests fail to account for psychological readiness, movement quality, and neuromuscular control under fatigue, all of which are crucial factors in mitigating reinjury risk [34]. Additionally, the traditionally adopted 6- to 9-month return-to-play criteria have been increasingly challenged, with growing evidence suggesting that time is merely one component of a much more complex rehabilitation framework [12]. This underscores the importance of progressive rehabilitation programs that are tailored to an individual’s functional and psychological status rather than arbitrary time-based milestones.

A key determinant of successful RTP is compliance with rehabilitation protocols [26]. Research has shown that 86% of fully compliant athletes return to their preinjury level of sport, compared with only 50–45% of those with partial or minimal compliance [26]. This highlights the necessity of individualized, high-quality rehabilitation programs that extend beyond basic strength and hop testing to include neuromuscular training, psychological assessments, and sport-specific conditioning [26].

RTP testing after ACLR should require re-evaluation to integrate a more comprehensive, evidence-based approach [32,35]. Rather than relying on rigid functional test criteria, clinicians and rehabilitation specialists should adopt a dynamic, individualized strategy that incorporates movement quality, psychological readiness, and patient-specific recovery trajectories [32,35]. Future research should focus on refining the RTP benchmarks to enhance their clinical utility and predictive accuracy, ultimately improving long-term outcomes for athletes post-ACLR.

***But what about time?***

For the majority of stakeholders, time is considered the primary criterion, followed by strength, hop, and other jump test. In a review of 209 studies, 85% reported the use of time as a criterion for RTP, with 42% relying on it as the sole criterion [32,35].

**Figure 2:**
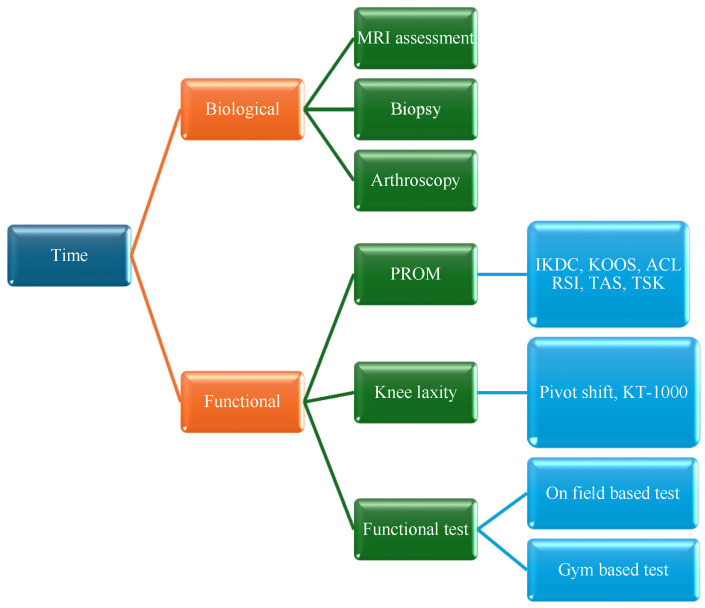
Representation of time as a combination of biological and functional factors.

We suggest interpreting time as a representation of two main components: biological time and functional time (Figure 2).

The first refer to the process of ligamentization, the second to the recovery of deficit after ACL rupture and reconstruction.

***But how can we assess healing?***

The free tendon grafts remain viable in the human knee after ACL reconstruction and undergo a process called ligamentization, where the graft progressively loses its tendon characteristics and acquires ligament-like properties [36]. While extensive data from animal studies exist, no ideal model has been established. Moreover, significant differences in healing timelines and necrosis patterns have been observed between animals and humans [36,37,38]. Some studies suggest that histologically mature grafts resemble native ACLs, but ultra-structural differences in collagen fibril distribution persist, preventing the full replication of a natural ACL [37,38].

During this process, the graft undergoes three distinct phases [37,38]:

1. Healing and Necrosis Phase—The host initiates an inflammatory response, leading to graft necrosis, particularly in the central region.
2. Remodeling Phase—The graft loses some of its mechanical properties, including the stiffness and collagen crimp pattern, while increasing its type III collagen content and undergoing cellular repopulation. At the same time, the host initiates an angiogenesis process.
3. Maturation Phase—The graft progressively acquires ligamentous properties, and the bone tunnel closes.

**Figure 3:**
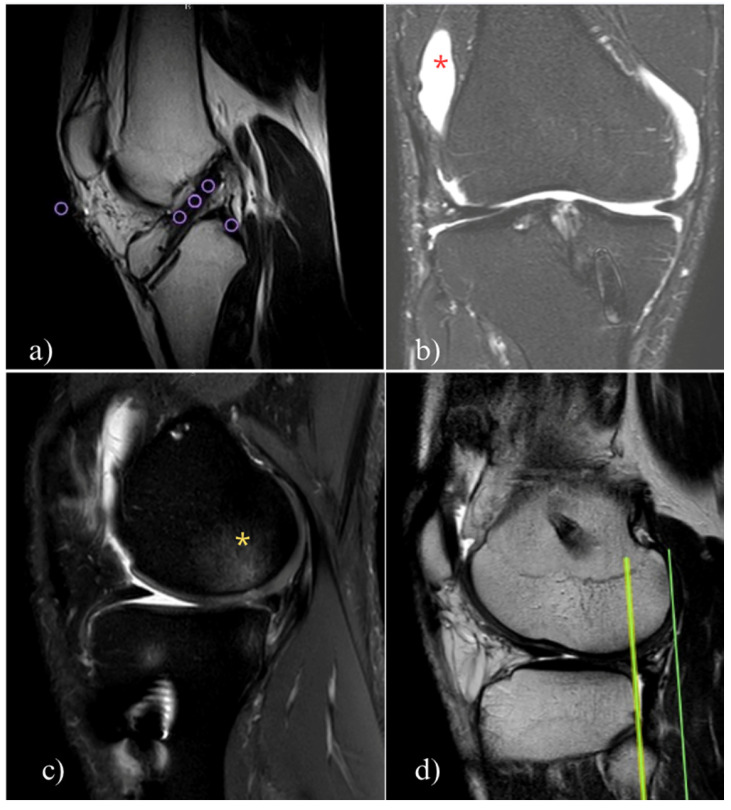
Common direct signal of graft maturity in MRI. (**a**) The signal–noise quotient is calculated by averaging the signal intensity from the distal, middle, and proximal grafts, subtracting the signal from the posterior cruciate ligament, and dividing it by the background signal. The purple circles indicate the region of interest for detecting the signal intensity. (**b**) Intra-articular swelling; the red asterisk indicates swelling. (**c**) Bone bruise of the posterior lateral femoral condyle; the yellow asterisk indicates the bone bruise in the posterior lateral femoral condyle. (**d**) Posterior femoral translation relative to the tibia; the dark green line indicates the femoral position relative to the tibial position (light green line).

Some evidence suggests that in some cases, ligamentization may remain incomplete even after 2 years, and certain clinical indicators derived from magnetic resonance imaging (MRI) may provide insights into this process. These indicators include the signal–noise quotient, bone bruise, intra-articular edema, intraligamentous signal hyperintensity, meniscus or cartilage damage and entirety during its course, the graft bending angle, tibial internal rotation, and anterior translation in relation to the femur [39,40,41] (Figure 3).

One of the most important tools for assessing graft healing is the signal-to-noise quotient, which compares the signal from the graft to that of the healthy posterior cruciate ligament and the background [42]. Various studies have correlated a low signal intensity from the graft with better maturation quality and improved knee function [41,43]. For example, in two different studies, a low signal of the graft for the T2 sequence demonstrated the ability to predict failure to RTP, with a sensitivity of 67.9% and specificity of 88.2%. Additionally, a low signal quotient of 0.078 ± 0.061 was associated with less than 1 mm of displacement on the KT-1000 test [41,43].

ACL graft maturation is a continuous process over the first two years post-surgery, with relevant inter-subject variability. Indeed, some MRI findings indicate that the graft signal does not fully reach the characteristics of a healthy ACL or posterior cruciate ligament, even at the two-year follow-up. At the same time, some studies show a good signal at 6 months in quadriceps and hamstring grafts [39,44,45,46]. The poorest MRI signal appears between 24 and 36 weeks post-surgery, reflecting a peak in remodeling activity [39]. The graft signal intensity increases at six months, remains significantly different from a native ACL, but progressively approximates native ACL characteristics at up to 24 months [40]. Notably, patients with a hypointense ACL graft signal at two years were more likely to return to their preinjury sport levels, suggesting that MRI could be useful for tracking graft maturation [43,45]. However, clinical assessments of knee function and stability remain essential for determining RTP readiness [42].

**Figure 4:**
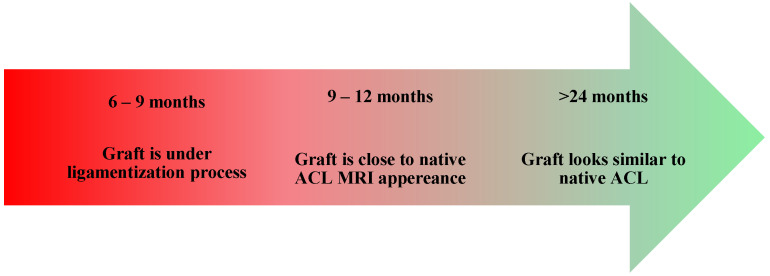
Timeline of ligamentization process based on MRI.

Despite MRI’s role as a noninvasive tool for monitoring graft changes, MRI signal-to-noise ratio (SNQ) variations do not reliably predict histologic remodeling stages, clinical outcomes, or functional recovery [42]. Additionally, the MRI-based graft maturity cannot accurately determine RTP timing in the first year after ACLR, making it an unreliable standalone assessment [47,48]. Functional and clinical evaluations, including proprioception, strength, and knee stability, remain more relevant than MRI findings in guiding RTP decisions [49]. Furthermore, variability in imaging techniques, scanner hardware, and acquisition protocols limits the reliability of SNQ as a universal indicator of graft healing (Figure 4). Some authors suggest a “two-year window” in which the graft and all the consequences of injury and surgery are resolved from a biological and functional perspective, but we must consider that most high-level athletes need to come back to the field earlier than recommended by the study [50].

Currently, due to the heterogeneity of acquisition and interpretation methods, MRI studies provide conflicting results regarding the graft maturation process and do not allow for a definitive determination of healing stagnation [38]. The gold standard for assessment remains biopsy; however, it is not a feasible examination for patients [38].

***What about functional deficit?***

As outlined above, there is great heterogeneity across studies regarding the type of rehabilitation and RTP test that should be used to determine athletes’ readiness to return to their sport [51]. In addition, most athletes show persistent deficits in certain kinetic variables such as the isokinetic/isometric strength and rate of force development (RFD) of hamstrings and quadricep muscles, vertical jump impulse and vertical ground reaction force, reactive strength (the less reactive strength index), and other kinematic variables such as reduction in knee flexion angle/increase in hip flexion during hops and jumps from 6 to 12 months after reconstruction [52,53,54,55,56,57,58,59,60].

One key aspect of the RTP test is the symmetry between the healthy and injured leg. It is important to note that achieving normal or increased symmetry in hop distance may sometimes mask compensatory strategies in joint mechanics, landing, or propulsion. Furthermore, estimating the capacity of the healthy leg could lead to an overestimation of the injured leg’s function due to strength or functional reductions during the postoperative rehabilitation period [53,54,61].

For this reason, it is crucial for practitioners to quantify not only outcomes such as the mean force, jump height, or hop distance but the neuromuscular strategies that are used to achieve this result as well. In addition, comparisons should be made with the contralateral leg, and, at the same time, with preinjury data of both legs, defined as the “estimated pre-injury capacity” (EPIC), and with similar healthy sport populations as a benchmark [59,60,61,62,63,64]. We recommend training the contralateral leg and continuing to use it as a reference for the ACLR leg [65].

For an accurate understanding of kinematic variables, the gold standard tools are triaxial force plates and 3D cameras. Nevertheless, due to high costs, limited availability in most clinical settings, and challenges in field applications, smartphone camera apps may represent a good and feasible alternative, providing insights into joint movement strategies, although they are not appropriate for quantifying the contribution of joint work to a task [66,67,68].

**Figure 5:**
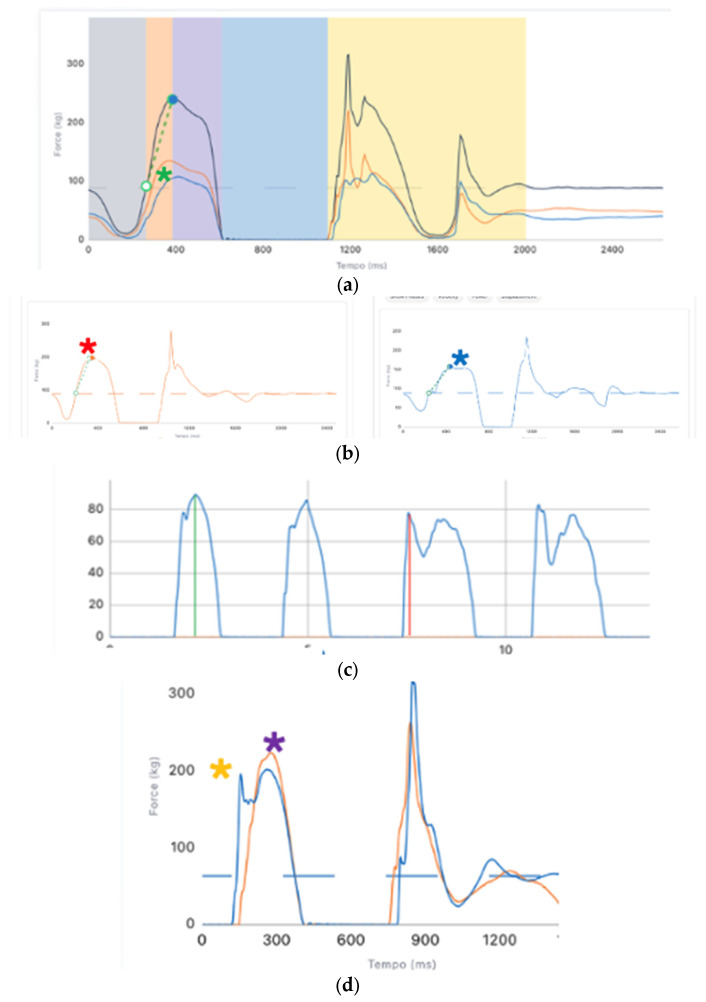
(**a**) Force-time curve of double countermovement jump (CMJ) show a shift of the load from the injured leg (blue curve) to the healthy leg (orange curve) during all phase of the jump. Green asterisk show difference in the force produced by two legs; (**b**) Force-time curve of a single leg CMJ show more eccentric- concentric mean force, peak force, impulse, jump height and power of the healthy leg (orange curve) respect to the injured one (blue curve). Red asterisk show an high level of eccentric and concentric vertical ground reaction force, blue asterisk a reduction in eccentric–concentric force production; (**c**) Force-time curve of single leg landings show in the first two curve (heathy leg) a well-accepted vGRF (vertical ground reaction force) (green line), in the third and fourth curve (injured leg) a stiff landing strategy with a reduction in vGRF (red line); (**d**) Force—time curve of a single leg vertical drop jump (DJ) show an optimal stretch shortening cycle in the orange curve (healthy) a non optimal strategies with less reactive strength on the injured leg (blue curve) After the first peak (purple asterisk) healthy leg start immediately an upward movement, in the second curve, after the first peak (orange asterisk) patient showed a loss of ground reaction force, suggest an impairment in stretch-shortening cycle.

**Figure 6:**
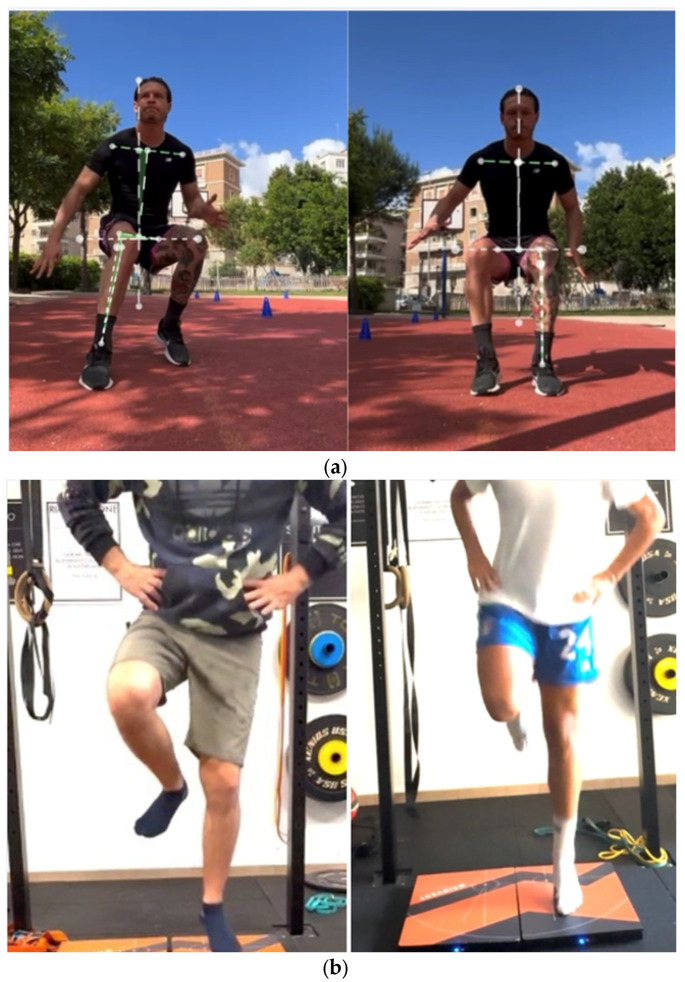

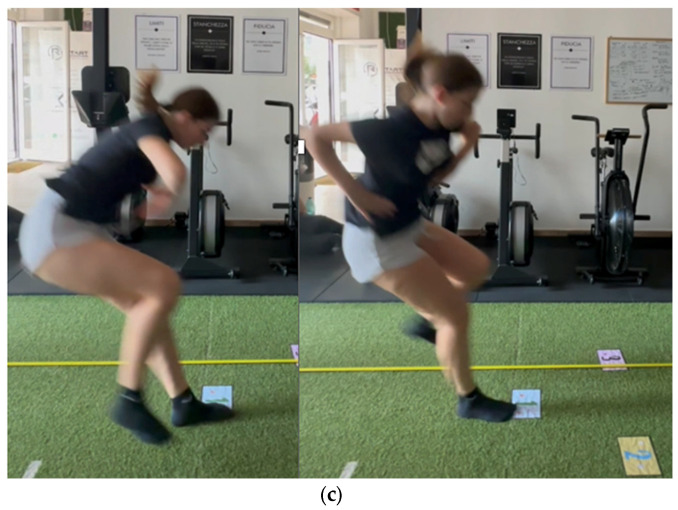
(**a**) A markerless motion analysis of a deceleration task shows increased knee valgus and trunk side flexion on the injured leg in the left image, while the healthy leg demonstrates optimal frontal plane strategies. (**b**) A kinematic analysis of a single-leg CMJ (left image) shows reduced terminal knee extension and maximal foot plantarflexion, whereas the right image shows no deficit in lower limb triple extension. (**c**) Single-leg hop–landing strategies: The left image (ACLR leg) depicts a suboptimal landing with increased hip and trunk flexion, whereas the right image (healthy leg) demonstrates an optimal landing with quadricep dominance.

Some examples of functional deficits at 8 months post-surgery are reported in Figure 5a–d and Figure 6a–c.

***How can we determine if an athlete is ready to return?***

There is no uniform consensus on this matter. One of the primary measures used to assess recovery during a task is the limb symmetry index (LSI). It is widely accepted that an optimal cutoff value for RTP should be above 90% symmetry in different tasks. However, the use of the LSI remains controversial, as research has shown that achieving >90% of LSI does not necessarily reduce the risk of reinjury, and, at the same time, this threshold if often not met by non-injured control subjects, suggesting that the natural variability in human motion may not be adequately represented by a 10% asymmetry margin [33,62]. One of the key aspects suggested by the authors is the concept of the “envelope of function”, which emphasizes the importance of a progressive load exposure that gradually prepares the limb for sport-specific demands, rather than basing RTP decisions on a single test [62,69]. We believe that tests should be used to assess deficits and function throughout the rehabilitation process, but they should not be used in isolation to determine a player’s readiness to return.

As previously mentioned, the successful completion of a specific rehabilitation program is likely a better indicator of readiness [26]. Compliance and adherence to the process are key milestones in the RTP continuum. The ability to reach loads that match the preinjury capacity is probably one of the most challenging and crucial aspects of this journey [13,70].

**Figure 7:**
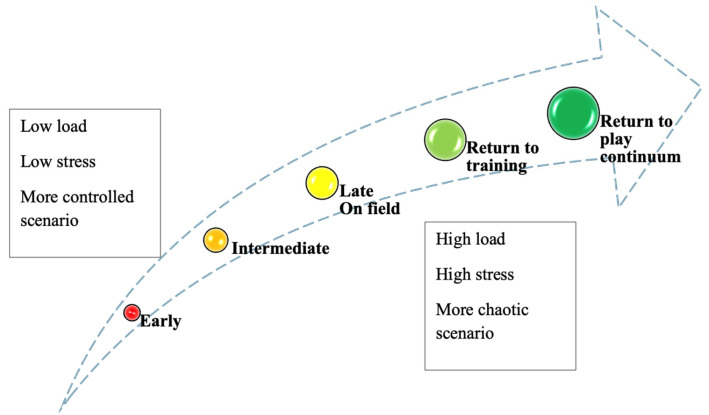
Progressive rehabilitation program. Each phase should focus on specific goals such as walking without crutches, initiating running, beginning on-field rehabilitation, and returning to team training.

The athlete should undergo a comprehensive rehabilitation process, progressing through the initial, intermediate, and late phases, followed by on-field rehabilitation, returning to training with and without restrictions, and, ultimately, full RTP [71,72] (Figure 7).

To summarize, the RTP process is a continuum of events in which key performance indicators (KPIs) help us better understand and assess functional recovery. At the same time, progressive exposure to sport-specific loads represents a critical component [13,54,73,74]. Examples of KPIs and RTP criteria are outlined in Table 1.

***The one-million-dollar question: when or how?***

In this scenario, time is not considered an RTP criterion. However, research suggests that the RTP timing may be correlated with the risk of reinjury, although the evidence on this topic remains conflicting.

The idea is that a prolonged period between ACLR and RTP promotes the ligamentization process, but the completion of this process is highly variable and not sufficiently verifiable by means of MRI assessment. The endpoint of ACL healing varies across studies, ranging from 1 to 4 years, with no well-established definition of healing (e.g., changes in crimp pattern, collagen composition, or graft continuity) [37].

Of course, a “better safe than sorry” approach is ideal for everyone, but waiting 12 to 24 months for RTP is not feasible for everyone, especially high-performance athletes.

Numerous studies indicate that a return to pivoting sports before 6 months post-surgery represents an unacceptable risk, whereas this risk appears to decrease after 9 months, especially in young, active individuals [75,76,77,78].

In contrast, some authors suggest that determining an optimal temporal cutoff to minimize the risk of reinjury is highly challenging due to the significant heterogeneity in study results [70]. This variability makes it difficult to provide accurate recommendations regarding the appropriate timing for returning to sport. Moreover, time alone is unlikely to be a standalone risk factor for reinjury [12,70,79]. Other factors, such as the lateral tibial slope, age, type of sport, and sex, appear to have a greater impact on the reinjury risk and should be carefully considered in return-to-sport decision making [12,70,79,80].

For this, we suggest categorizing the RTP timeline into four groups:

- RTP <6 months;
- RTP 6–9 months;
- 9–12 months;
- >12 months.

Once we consider time, we must also account for functional recovery within each category.

Time alone does not provide sufficient information about a patient’s recovery; the “how” must be integrated into the decision making process.

The question is therefore clear: How many patients can fully recover their function and complete a full rehabilitation/training program in less than six months?

Probably fewer than we think. If we consider the entire population who is affected by ACL injuries, not everyone can rehabilitate 3 to 5 days a week, attend double sessions daily, or access top-tier devices for treatment and assessment. For example, Piccinini et al. [81], reported a high RTP rate in soccer players (elite and amateur) who completed a five-stage on-field rehabilitation (OFR) program, aligning their training with team GPS data before returning to full training [81]. Based on our personal clinical experience, we would also like to point out that excessive loading during rehabilitation may expose the knee joint to unaccustomed stresses, thus increasing the risk of postoperative complications, and that this type of rehabilitation is not accessible to everyone.

The notion that 5, 6, or 9 months is sufficient to recover from these deficits applies only to a subset of ACL-injured patients, specifically those who have the opportunity to expose themselves to workloads earlier and more comprehensively than the general non-elite-athlete population.

Currently, the literature remains conflicting regarding the risk of reinjury before six months [12,70,78,79]. Therefore, the key question is the following: Have we ensured that the athletes have fully recovered from their injuries and been adequately exposed to sport-specific loads?

**Figure 8:**
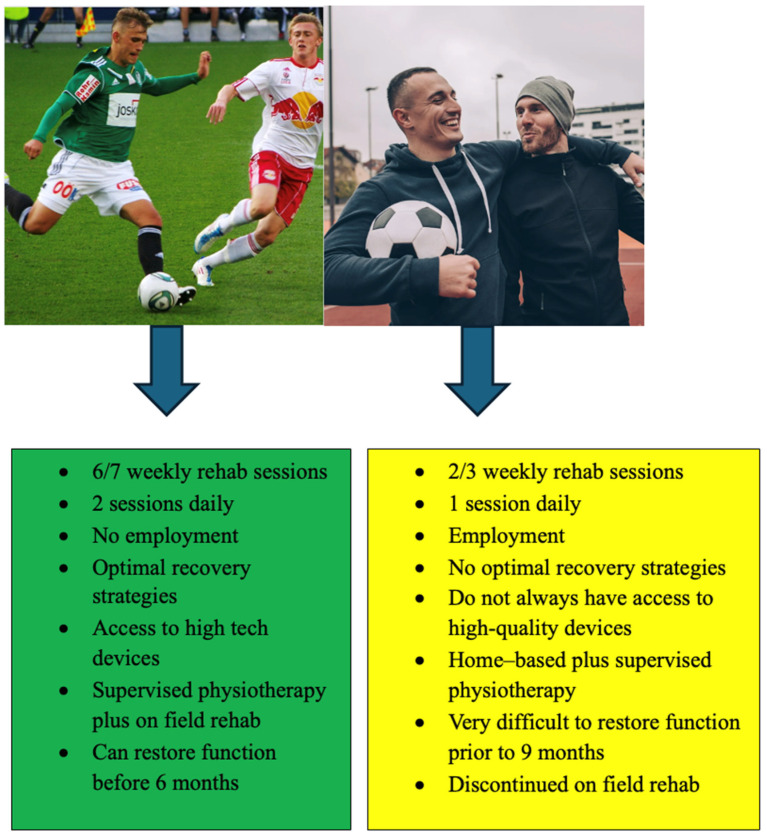
Differences between two main athlete categories: elite (left) and non-professional athletes (right).

Furthermore, we must consider two distinct categories of patients: professional athletes, who have extensive rehabilitation resources, and non-professional ones, whose rehabilitation opportunities are significantly more limited (Figure 8).

***When evaluating time and function, the key question is, “Is the athlete truly ready to return without functional deficits?”***

**Figure 9:**
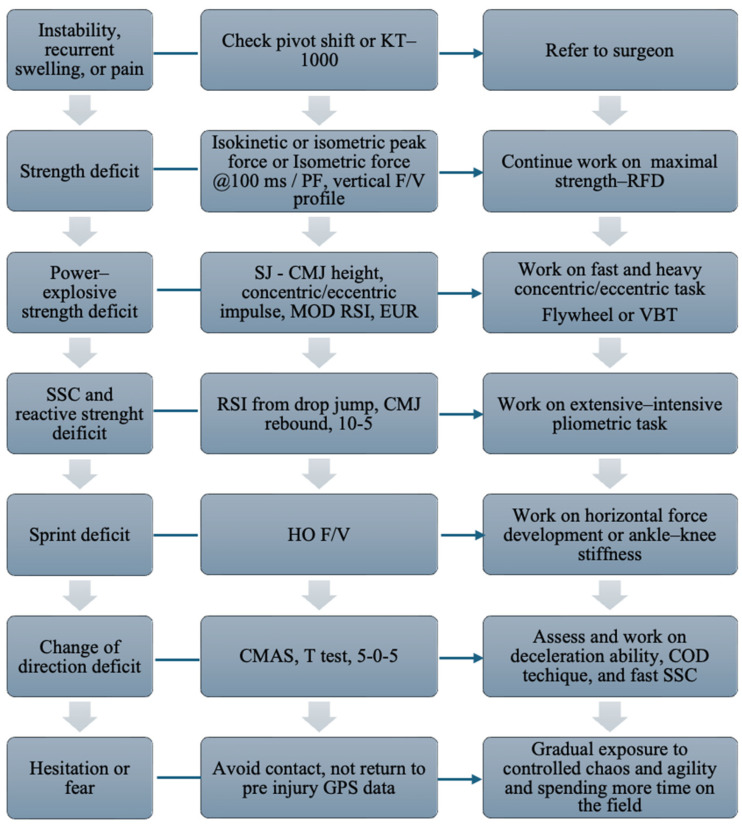
Overview of the assessment and treatment of functional deficits after ACLR (anterior cruciate ligament reconstruction). SJ: squat jump; CMJ: countermovement jump; SSC: stretch-shortening cycle; COD: change of direction; MOD RSI: reactive strength index modified; RSI: reactive strength index; HO F/v: horizontal force velocity profile; V F/v: vertical force velocity profile; CMAS: change of direction assessment score; T test: agility T test; VBT: velocity-based training; EUR: eccentric utilization ratio; RFD: rate of force development, isometric force @100 ms/PF; vertical: ratio between force at 100 ms and peak force; PF: peak force.

An example of how to assess and treat functional deficits is reported in Figure 9.

If the answer is yes, then time may not be the most critical factor, as long as the return occurs within the 6- to 9-month window. However, the RTP process should be guided by the Strategic Assessment of Risk and Risk Tolerance (StARRT) framework. The StARRT framework incorporates three key components in the RTP decision making process:

- Tissue Health, which evaluates the injured tissue’s ability to tolerate stress;
- Tissue Stress, which assesses the mechanical load that is imposed by sports activity and the effectiveness of protective measures;
- Risk Tolerance Modifiers, which consider external factors such as the competitive level, financial incentives, and psychological well-being.

This structured and transparent approach allows clinicians to balance medical risks with the athlete’s overall well-being, facilitating safer and more informed RTP decisions [82].

***Analyses of examples:***

***(Example 1)***

Consider the following scenario: A 24-year-old elite-level football player, a starting forward, underwent ACL reconstruction surgery seven months ago. He has completed a structured rehabilitation program, fully recovered from his injuries, reports psychological readiness, and exhibits no signs of knee instability or discomfort. Both the medical and athletic staff have approved his return play.

At this stage of the season, the team is competing in the Champions League semifinals, and due to injuries, no other starting forwards are available, forcing the coach to field an inexperienced youth player. Additionally, the athlete is nearing a contract renewal, adding further complexity to the decision.

While this situation could be considered high-risk based on the timeline and player profile, a thorough risk–benefit analysis may lead both the medical staff and the athlete to accept an accelerated return to play, balancing competitive demands with the player’s physical readiness.

***(Example 2)***

Consider the following scenario: A 21-year-old football player, a non-starter, underwent ACL reconstruction 8.5 months ago. He plays as a wide midfielder, and his team is currently mid-table halfway through the season. There are other players available in his position, and he is not nearing a contract renewal.

Despite the time elapsed since surgery, he has not fully recovered from his injuries, continues to experience difficulties handling training loads, and exhibits deficiencies in jump performance.

In this case, the potential risks outweigh the benefits, as the player is neither a key figure in the squad nor involved in a high-stakes competitive scenario. Given his ongoing physical limitations, a “better safe than sorry” approach is advisable, prioritizing full functional recovery before considering a return to play.

If the athlete returns before 6 months, there is a significantly higher risk that he has not fully recovered. In this case, the ACL may still be undergoing a decline in its mechanical properties, increasing the likelihood of reinjury [76].

**Figure 10:**
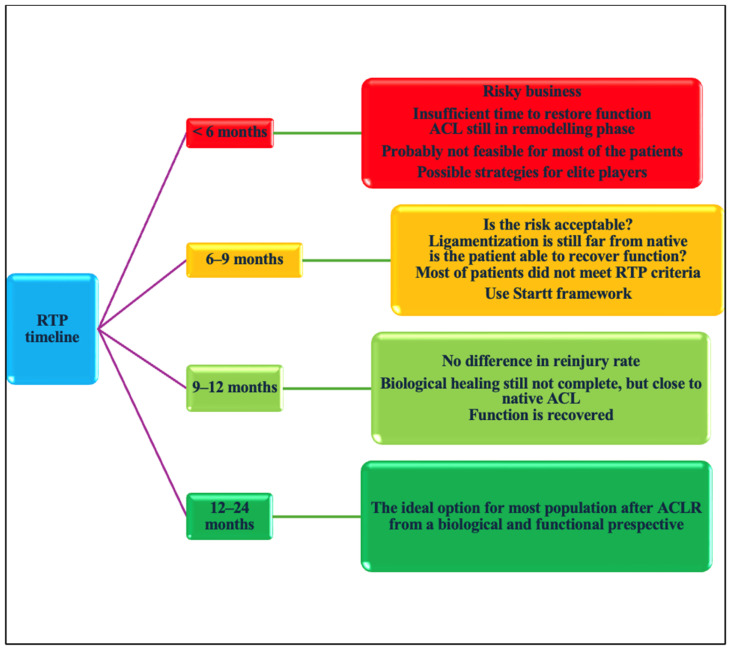
Framework for RTP based on time and functional recovery.

The flowchart presented below illustrates the framework for RTP (Figure 10).

In this complex context, the time between injury and surgery is often overlooked, despite being positively correlated with a return to performance in level 1 sport 12 months after surgery [83].

## 2. Conclusions

The RTP pathway following ACL injury is a long and structured process, guided by the achievement of progressive goals, assessed through periodic evaluations [70]. A goal-oriented rehabilitation approach, rather than a time-based one, is fundamental to decision making throughout recovery [84]. However, time remains a relevant factor in guiding specific milestones, such as returning to running or initiating change-of-direction activities, serving as a supporting parameter alongside functional assessments throughout a progressively increasing training intensity [71].

In this context, the role of time in the reinjury risk remains controversial, as the current literature does not provide a definitive safe temporal cutoff. Instead, evidence increasingly supports a “how” rather than “when” approach to RTP [70]. It is crucial to recognize that functional deficits may persist well beyond the conventional nine-month timeframe, and an RTP before this period is often unsuitable for a large proportion of athletes.

Nevertheless, in specific conditions, when clinical and functional benchmarks are met, and risk stratification is applied, an earlier RTP (<9 months) may be feasible.

Time alone cannot restore deficits, so we strongly recommend continuously assessing the patient throughout the rehabilitation process to monitor the progression of deficits and detect any potential deconditioning of the uninjured leg. Beyond the use of criteria, it is crucial to adopt an approach that progressively exposes the patient to increasingly complex and unpredictable scenarios, both in the gym and on the field, in order to prevent excessive disparities between training and cognitive loads and the competitive demands of the sport [85,86]. The current evidence remains uncertain regarding the optimal timing for RTP, although over time, the concept of waiting at least nine months has gained traction. However, the heterogeneity of studies—including differences in athletic populations (often compared despite their variability), patient age, surgical timing, economic resources, and access to intensive rehabilitation services—prevents definitive conclusions about when a patient is at a higher or lower risk of reinjury.

Future research should aim to refine individualized RTP criteria, integrating objective biomechanical, neuromuscular, and psychological markers to optimize both performance outcomes and long-term joint health.

## Figures and Tables

**Table 1:** Key performance indicators (KPIs) to guide RTP tests [13,54,73,74].

KPI	Value
Knee laxity–swelling–pain	Absent
PROMs (IKDC, KOOS, ACL-RSI)	Normalized
Isokinetic strength	>90% flexion and extension 60–180°/s LSI
Single-leg CMJ impulse and height	>90% LSI
Single-leg DJ RSI and height	>90% LSI
Hop distance (battery)	>90% LSI
Side hop repetition	>90 LSI
Jump–hop biomechanics	Symmetry value for moments, angles, and work in vertical and horizontal jumps, especially in sagittal and frontal planes at hip, knee, and ankle
Double-leg CMJ and DJ	Symmetry in vGRF
Running–sprinting profile (1080 sprint or Myjump ®) and biomechanics	Same vGRF; same knee, hip and ankle angle; no trunk side flexion or lack of pelvis frontal plane control Preinjury horizontal F/V profile
Running sprinting curriculum	Preinjury velocity in linear, curvilinear, and multidirectional movements
Repeated sprint ability test index	<3%
Agility test (T-test, Illinois)	Normative value
Adherence to a structured rehabilitation program	More than 50 sessions, more than 30 sessions on field, more than 3 sessions per week
45′ Game simulation—preinjury training load	GPS data

KPI: key performance indicator; PROMS: patient-reported outcomes; IKDC: International Knee Documentation Committee Subjective Knee Form; KOOS: Knee Injury and Osteoarthritis Outcome score; ACL-RSI: Anterior Cruciate Ligament—Return to Sport after Injury Scale; LSI: limb symmetry index; CMJ: countermovement jump; DJ: drop jump; F/V: force velocity profile; vGRF: vertical ground reaction force.

## Data Availability

No new data were created.

## Abbreviations

The following abbreviations are used in this manuscript:

ACL	anterior cruciate ligament
ACL-RSI	Anterior Cruciate Ligament—Return to Sport after Injury Scale
ACLR	anterior cruciate ligament reconstruction
CMAS	change of direction assessment score
CMJ	countermovement jump
COD	change of direction
DJ	drop jump
EPIC	estimated preinjury capacity
EUR	eccentric utilization ratio
F/V	force velocity profile
HO F/v	horizontal force velocity profile
IKDC	International Knee Documentation Committee Subjective Knee Form, isometric force @100 ms/PF, vertical ratio between force at 100 ms and peak force
KOOS	Knee Injury and Osteoarthritis Outcome score
KPI	key performance indicator
LSI	limb symmetry index
MOD RSI	reactive strength index modified
MRI	magnetic resonance imaging
PF	peak force
PROMS	patient-reported outcomes
RFD	rate of force development
RSI	reactive strength index
RTP	return to play
SJ	squat jump
SSC	stretch-shortening cycle
SNQ	signal–noise quotient
T test	agility T test
V F/v	vertical force velocity profile
VBT	velocity-based training
vGRF	vertical ground reaction force
